# Clinical therapeutic effects of lidocaine combination with flurbiprofen axetil for reducing propofol-induced pain in adults: A protocol for systematic review and meta-analysis

**DOI:** 10.1097/MD.0000000000023844

**Published:** 2020-12-24

**Authors:** Weiqiang Sun, Jinfen Yu, Gang Lu, Xiaofeng Ye, Jun Fu

**Affiliations:** Pain Management, Maternal and Child Health Hospital of Hubei Province, Wuhan, Hubei, PR China.

**Keywords:** effectiveness, flurbiprofen axetil, lidocaine, meta-analysis, pain, propofol

## Abstract

**Background:**

Pain on injection is a well-recognized adverse event of propofol administration for the stimulation of general anesthesia. Pre-treatment with lidocaine or flurbiprofen axetil has proven to be effectual in the reduction propofol-induced pain in adults. Nonetheless, only few studies have evaluated the clinical therapeutic effects of lidocaine combination with flurbiprofen axetil to prevent pain on injection of propofol. The current study aims to evaluate the clinical therapeutic effects of lidocaine combination with flurbiprofen axetil to reduce pain on injection of propofol among adult patients.

**Methods:**

The literature search will be conducted from their inception to November 2020 from MEDLINE, EMBASE, Web of Science, and Cochrane Library databases without date or geographical restrictions. However, language will be restricted to publications in English and Chinese. Two authors will independently screen abstracts and titles of all papers to determine whether to include or exclude them. The authors will also study characteristic and outcomes of data extraction and carry out risk of bias assessment. We plan to use either a fixed-effects or random-effects model to estimate the risk ratios (RR) or mean difference (MD) or standardized mean difference (SMD) together with 95% confidence interval (CI).

**Results:**

This study will provide high-quality evidence for the clinical therapeutic effects of lidocaine combination with flurbiprofen axetil for reducing pain on injection of propofol in adult patients.

**Conclusion:**

This study will summarize current evidence for the management of pain on injection of propofol in adult patients and provide guidance for both intervention and future research.

**Ethics and dissemination:**

Since no data collection will be involved, there is no need for an ethics approval.

**Registration number:**

November 17, 2020.osf.io/72tpj/. (https://osf.io/72tpj/)

## 1 Introduction

Propofol is usually utilized in painless endoscopy anesthesia due to its benefits, such as the rapid onset and its relation with early recovery. Pain on injection, however, is a well-known adverse even usually associated with administration of propofol for the stimulation of general anesthesia.^[1,2]^ Pain induced by propofol injection occurs more frequently in untreated patients with an incidence ratio from 28 to 90%.^[3,4]^ Various pharmacological interventions have been tried out to try and reduce pain on injection of propofol, socially among adult patients, including pre-treatment with different medications such as dexmedetomidine, metoclopramide, lidocaine, and flurbiprofen axetil.^[5–8]^ While pretreatment using dexmedetomidine can significantly reduce propofol-induced injection pain efficiently and securely, its lengthy onset is not suitable for clinical utilization. Therefore, flurbiprofen, which is a non-selective cyclooxygenase inhibitor can be used clinically as non-steroidal anti-inflammatory drugs (NSAID). In particular, flurbiprofen axetil is regarded as an injectable product of flurbiprofen for post-operative pain control.^[8,9]^ However, no studies have evaluated the clinical therapeutic effects of lidocaine combination with flurbiprofen axetil to prevent pain on injection of propofol. Thus, the current study will explore the best available evidence, focusing on clinical therapeutic effects of lidocaine combination with flurbiprofen axetil to reduce pain on injection of propofol among adult patients.

## 2 Methods

We have registered this protocol on the Open Science Framework (OSF, http://osf.io/), and the registration number was 10.17605/OSF.IO/72TPJ. The study will be reported in accordance with guidelines of the Preferred Reporting Items for Systematic Reviews and Meta-Analysis Protocol (PRISMA-P) statement.

### 2.1 Criteria for inclusion in the study

#### 2.1.1 Types of studies

Randomized controlled trials (RCTs) evaluating the clinical therapeutic effects of lidocaine combination with flurbiprofen axetil will be included to ascertain their impact to prevent pain on injection of propofol.

#### 2.1.2 Types of participants

Participants to be considered for this study will include adult patients aged above 18 years who were administered propofol intravenously.

#### 2.1.3 Types of interventions

1. The experimental groups
  In the experimental groups, any RCTs study that utilize lidocaine combination with flurbiprofen axetil as intervention will be included in the present study.
2. The control groups
  In the control groups, any RCTs study that utilize any treatments with lidocaine alone or flurbiprofen axetil alone or any another treatment.

#### 2.1.4 Types of outcome measures

The primary outcomes will include incidences of propofol-induced injection pain. The secondary outcomes will include the gravity of propofol-induced injection pain, adverse outcomes, including thrombophlebitis, cardiac arrhythmia, hypoxemia, hypotension, allergic reaction, vital signs, and patient satisfaction.

### 2.2 Search methods for identification of studies

#### 2.2.1 Electronic searches

We intend to perform a literature review from inception to November 2020 in MEDLINE, EMBASE, Web of Science, and Cochrane Library database without date or geographical restrictions. However, language will be restricted to publications in English and Chinese. The following search terms: propofol∗, lidocaine∗, flurbiprofen∗, adult∗, “injection pain”, “RCT∗”, “randomized controlled trial”, “randomised controlled trial”, and randomly∗ will be combined using “AND” or “OR” to identify relevant studies.

#### 2.2.2 Searching other resources

ClinicalTrials.gov (https://clinicaltrials.gov/) will be searched and the reference lists of all the main articles will be checked to obtain extra references.

### 2.3 Data collection and analysis

#### 2.3.1 Selection studies

**Figure 1:**
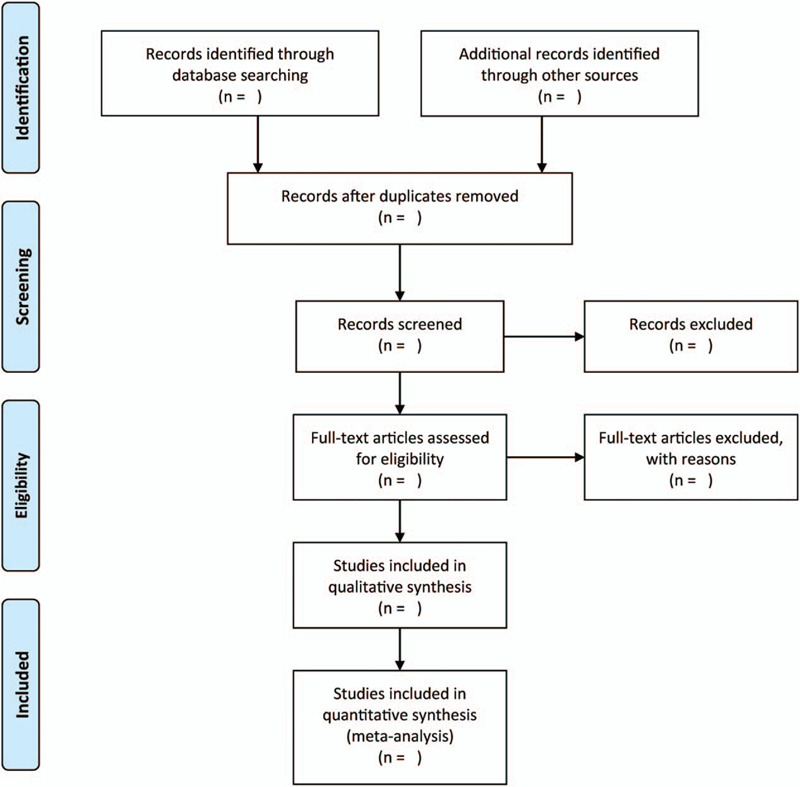
The research flowchart.

We will use 2 independent authors to screen the titles and abstracts of papers obtained from the literature search. We will obtain full-texts and reports of all possibly works. Accordingly, 2 independent authors will screen the papers and consider whether they should be included or excluded. Any disagreements between the authors will be addressed through discussion or by consulting a third author when needful. We will illustrate our selection process in Figure 1.

#### 2.3.2 Data extraction and management

After that, 2 independent authors will use a particular data extraction form for extracting outcome data from the included studies. The following data will be extracted: the general features of included studies, the sample size, study setting, study methods, the types, and general characteristics of participants, all outcome measures, and interventions and comparisons. Any disagreements between the authors will be addressed through discussion or by consulting a third author when needful.

#### 2.3.3 Assessment of risk of bias in included studies

Two independent authors will use the Cochrane Collaboration tool to assess the methodological quality of selected studies. There are seven domains and each one is further evaluated as “Low risk”, “High risk”, or unclear. Any disagreements between the authors will be addressed through discussion or by consulting a third author when needful.

#### 2.3.4 Measures of treatment effect

Results will be expressed as risk ratio (RR) together with 95% confidence intervals (CI) for all dichotomous results of the included studies. We will express results as mean difference (MD) or standardized mean difference (SMD) together with 95% CI for all continuous outcomes.

#### 2.3.5 Assessment of heterogeneity

We will use the *I*^2^ statistic to evaluate heterogeneity. We will plan to regard a level of heterogeneity above 50% as substantial heterogeneity, and then the random-effects model will be utilized to estimate the results.

#### 2.3.6 Assessment of reporting biases

We intend to establish and explore the funnel plots to determine potential publication bias where we can pool over 10 trials.

#### 2.3.7 Sensitivity analysis

We plan to perform a sensitivity analysis to evaluate the robustness of our findings if we identify sufficient studies.

## 3 Discussion

This study will provide an up-to-date assessment of the clinical therapeutic effects of lidocaine combination with flurbiprofen axetil to reduce pain on injection of propofol in adult patients. We consider that this study will focus on examining the clinical therapeutic effects of lidocaine combination with flurbiprofen axetil to treat adult patients with propofol-induced pain. Although several studies have proved the efficiency of lidocaine in prevention of pain on injection of propofol in adult patients, the use of lidocaine combination with flurbiprofen axetil to treat adult patients with pain on injection of propofol remains undiscovered. Therefore, the present study will systematically explore the clinical therapeutic effects of lidocaine combination with flurbiprofen axetil to prevent pain on injection of propofol in adult patients. The expected findings will provide recommendation for the treatment of adult patients with pain on injection of propofol.

## Author contributions

**Conceptualization:** Weiqiang Sun, Xiaofeng Ye, Jun Fu.

**Data curation:** Weiqiang Sun, Jinfen Yu, Gang Lu.

**Formal analysis:** Weiqiang Sun, Jun Fu.

**Funding acquisition:** Jinfen Yu, Xiaofeng Ye.

**Investigation:** Jinfen Yu, Jun Fu.

**Methodology:** Gang Lu.

**Project administration:** Jinfen Yu, Gang Lu.

**Resources:** Jinfen Yu, Gang Lu.

**Software:** Weiqiang Sun, Jinfen Yu.

**Supervision:** Gang Lu, Xiaofeng Ye.

**Validation:** Weiqiang Sun, Xiaofeng Ye.

**Visualization:** Jinfen Yu, Gang Lu, Jun Fu.

**Writing – original draft:** Weiqiang Sun, Xiaofeng Ye, Jun Fu.

**Writing – review & editing:** Weiqiang Sun, Xiaofeng Ye, Jun Fu.

## References

[1] Macario A Weinger M Truong P Which clinical anesthesia outcomes are both common and important to avoid? The perspective of a panel of expert anesthesiologists. Anesth Analg 1999;88:1085–91. 10320175 10.1097/00000539-199905000-00023

[2] Ji L Sun W Lan Y Dexmedetomidine for prevention of propofol injection pain upon induction of anesthesia: a meta-analysis. Eur J Clin Pharmacol 2020;76:1103–10. 32385544 10.1007/s00228-020-02889-x

[3] Mangar D Holak EJ Tourniquet at 50 mm Hg followed by intravenous lidocaine diminishes hand pain associated with propofol injection. Anesth Analg 1992;74:250–2. 1731546 10.1213/00000539-199202000-00014

[4] Picard P Tramèr MR Prevention of pain on injection with propofol: a quantitative systematic review. Anesth Analg 2000;90:963–9. 10735808 10.1097/00000539-200004000-00035

[5] Li X Chen CJ Tan F Effect of dexmedetomidine for attenuation of propofol injection pain in electroconvulsive therapy: a randomized controlled study. J Anesth 2018;32:70–6. 29127492 10.1007/s00540-017-2430-3

[6] Euasobhon P Dej-Arkom S Siriussawakul A Lidocaine for reducing propofol-induced pain on induction of anaesthesia in adults. Cochrane Database Syst Rev 2016;2:Cd007874. 26888026 10.1002/14651858.CD007874.pub2 PMC6463799

[7] Polat R Aktay M Ozlü O The effects of remifentanil, lidocaine, metoclopramide, or ketamine pretreatment on propofol injection pain. Middle East J Anaesthesiol 2012;21:673–7. 23265029

[8] Zhang L Zhu J Xu L Efficacy and safety of flurbiprofen axetil in the prevention of pain on propofol injection: a systematic review and meta-analysis. Med Sci Monit 2014;20:995–1002. 24935068 10.12659/MSM.890102 PMC4070992

[9] Mikawa K Nishina K Maekawa N Dose-response of flurbiprofen on postoperative pain and emesis after paediatric strabismus surgery. Can J Anaesth 1997;44:95–8. 8988832 10.1007/BF03014332

